# Hereditary α-tryptasemia; a review of mechanisms linking α-tryptase gene dosage to intestinal homeostasis and immunopathology

**DOI:** 10.3389/falgy.2026.1783914

**Published:** 2026-04-14

**Authors:** Ilaria M. Simeone, Michelle Galeas-Pena, Katelyn White, Brandy Sullivan, Alexandra Morelli, Jocelyn A. Silvester, Liza Konnikova, Amelie Therrien, Sarah C. Glover

**Affiliations:** 1 Department of Medicine, Section of Gastroenterology, Tulane University, New Orleans, LA, United States; 2 Celiac Center, Department of Gastroenterology, Beth Israel Deaconess Medical Center, Harvard Medical School, Boston, MA, United States; 3 Division of Gastroenterology, Hepatology and Nutrition, Boston Children Hospital, Boston, MA, United States; 4 Departments of Pediatrics, Immunobiology and Obstetrics, Gynecology and Reproductive Sciences, Yale University School of Medicine, New Haven, CT, United States

**Keywords:** gastrointestinal tract, hereditary α-tryptasemia, mast cells, tryptase, disease modification, gene dosage, inflammatory bowel disease, celiac disease

## Abstract

Hereditary α-tryptasemia (HαT) is a genetic trait characterized by increased *TPSAB1* copy number. Identified in 2015, the HαT trait impacts approximately 4%–6% of individuals of European ancestry and manifests with core clinical features in one-third of individuals who test positive for the genetic trait. HαT represents a natural human model of α-tryptase overexpression which can be leveraged to better and more comprehensively understand tryptase and mast cell (MC) biology at the tissue level. In this review, we synthesize emerging evidence demonstrating that HαT is a clinically significant modifier of disease in the gastrointestinal (GI) tract. We summarize findings demonstrating that HαT impacts small intestinal immunopathology even in the absence of overt GI pathology. In celiac disease, coexisting HαT is associated with increased duodenal MCs and persistent GI symptoms (diarrhea, bloating, abdominal pain) despite a gluten-free diet. We also review emerging data indicating that HαT may act as a disease modifier in inflammatory bowel disease (IBD); increased α-tryptase gene dosage is associated with intestinal MC activation and increased expression of MRGPRX2. These changes may amplify MC–mediated inflammatory pathways within the intestinal mucosa and contribute to the complexity of immune signaling traditionally attributed to T-cell–driven inflammation in IBD. Taken together, emerging modern cellular and molecular biology evidence suggests that the natural overexpression of α-tryptase in HαT alters MC behavior and GI intestinal immunopathology, thereby modifying disease outcomes across a spectrum of GI illnesses.

## 1 Introduction

### 1.1 History and biology of mast cells

**Figure 1:**
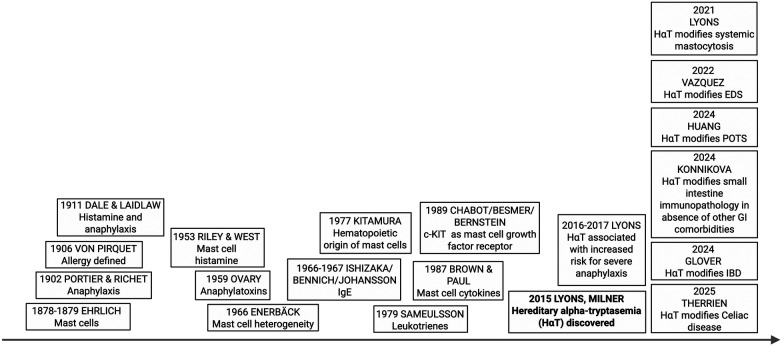
History of mast cell research. The discovery of the genetic trait associated with HαT has led to an explosion of mast cell-related research over the last 10 years. The timeline above depicts mast cell discoveries from 1878-current. Adapted from Blank et al. Allergy. 2013*.*

Mast cells (MCs) were first identified in the late 19th century. During the early to mid-20th century, the central role of MCs in anaphylaxis, histamine release, and allergic disease was further elucidated. Subsequent discoveries revealed that MCs are a heterogeneous population of hematopoietic origin that release cytokines, participate in IgE-mediated reactions, and require c-KIT signaling for differentiation. Importantly, MCs are long-lived, tissue-resident immune cells that can exert sustained effects on local tissues. The discovery of hereditary α-tryptasemia (HαT) in 2015 marked a significant inflection point in our understanding of MC biology. After more than two decades during which the clinical implications of elevated basal serum tryptase (BST) remained poorly understood, this discovery renewed interest in the role of MC-derived tryptases in human disease and clinical phenotypes (Figure 1). Of note, human MCs and tryptases are evolutionarily distinct from those of other species, underscoring the importance of further understanding how HαT influences human tissues (1, 2).

The purpose of this review is to synthesize current evidence demonstrating that HαT, a natural model of α-tryptase overexpression, modifies MC behavior and disease response in the human intestine even in the absence of other pathologies such as celiac disease (CeD) and inflammatory bowel disease (IBD).

### 1.2 Mast cell activation and the duodenal mucosal immune system in the healthy gut

MCs are hematopoietic stem cell-derived immune cells resident in mucosal and connective tissues that contribute to both innate and adaptive immune responses. Consequently, they play a key role in host defense, allergic inflammation, and maintenance of tissue homeostasis (3, 4).

MC activation can occur through both IgE-dependent and IgE-independent pathways. In classic allergic responses, antigen-specific IgE bound to the high-affinity Fc*ε*RI receptor on the MC surface is crosslinked by multivalent antigen, triggering intracellular signaling cascades that culminate in degranulation and mediator release (3). In addition to IgE, MC degranulation can also be activated by complement fragments, cytokines, neuropeptides, and engagement of receptors such as Mas-related GPCR-X2 (MRGPRX2) (5–10). MRGPRX2 is expressed predominantly on human MCs and is involved in non-IgE mediated, “pseudoallergic” hypersensitivity reactions. It is distinct from the high-affinity IgE receptor (Fc*ε*RI) which mediates classic IgE-dependent reactions (6). MRGPRX2 is mainly expressed in connective tissue MCs and intestinal MCs (5, 11). MRGPRX2 has been implicated in several allergic and inflammatory diseases including drug-induced anaphylaxis, asthma, urticaria, neurogenic inflammation, pruritus, and pain (12). Upon degranulation, MCs rapidly release a broad repertoire of preformed mediators, including histamine, proteoglycans, cytokines, and neutral proteases such as tryptases and chymases (4, 13, 14). MCs contain substantially higher amounts of tryptase than basophils; early quantitative analyses estimated approximately 12 pg of tryptase per MC compared with approximately 0.046 pg per basophil, corresponding to roughly a 250–300-fold difference (15). As a result, MC–derived tryptases represent major downstream effectors of MC activation and can influence epithelial barrier function, neuroimmune signaling, and inflammatory responses within the intestinal mucosa (14, 16, 17).

MC activation can be homeostatic or pathogenic (18). The mucosal immune system must continuously balance tolerance to innocuous environmental antigens, such as dietary components and commensal microbiota, with protection against potentially harmful pathogens. As the first part of the small intestine, the human duodenum is a critical site for nutrient absorption and for antigen processing by innate and adaptive immune cells, including MCs. As such, the duodenum represents an important interface for the study of MCs and their behavior within this tightly regulated immune environment. In the intestinal mucosa, duodenal MCs participate in barrier regulation, neuroimmune signaling, and coordination of epithelial and myeloid cell responses, positioning them as central contributors to the maintenance of mucosal immune equilibrium. When this balance is disrupted, duodenal MCs are also involved in the development of GI symptoms and chronic inflammatory disease.

Until recently, the cellular architecture of the human small intestinal mucosa, particularly during early life, has been poorly defined. Recent single-cell transcriptomic analysis of the pediatric duodenum across diverse ancestries has provided new insights on MC behavior in the healthy GI tract. In a diverse cohort of 88 children, mucosal MCs were identified by their high expression of MC granule genes including *CPA3*, *TPSB2*, and *TPSAB1*, as well as elevated expression of the surface marker *KIT* and *HDC* (enzyme required for histamine synthesis), consistent with established transcriptional signatures used to identify MC in mucosal tissues. Notably, despite their immune lineage, mucosal MCs have high transcriptional similarity to intestinal epithelial Tuft cells. Both of these cell types participate in Type 2 immunity. This analysis also revealed two distinct MC clusters, active and resting (50% each), in the healthy pediatric duodenum. The active cluster demonstrated upregulation of genes associated with MC degranulation including *SIGLEC6*, *CDK15*, *ST8SIA6*, *ENPP4*, *JAK1*, *STAT3*, and *GCSAML* (19). Interactions between active and resting MC clusters may be important in the maintenance of mucosal immune homeostasis (20).

## 2 Hereditary α-tryptasemia (HαT): definition, genetics, and clinical Spectrum

### 2.1 Genetic basis and clinical penetrance

**Figure 2:**
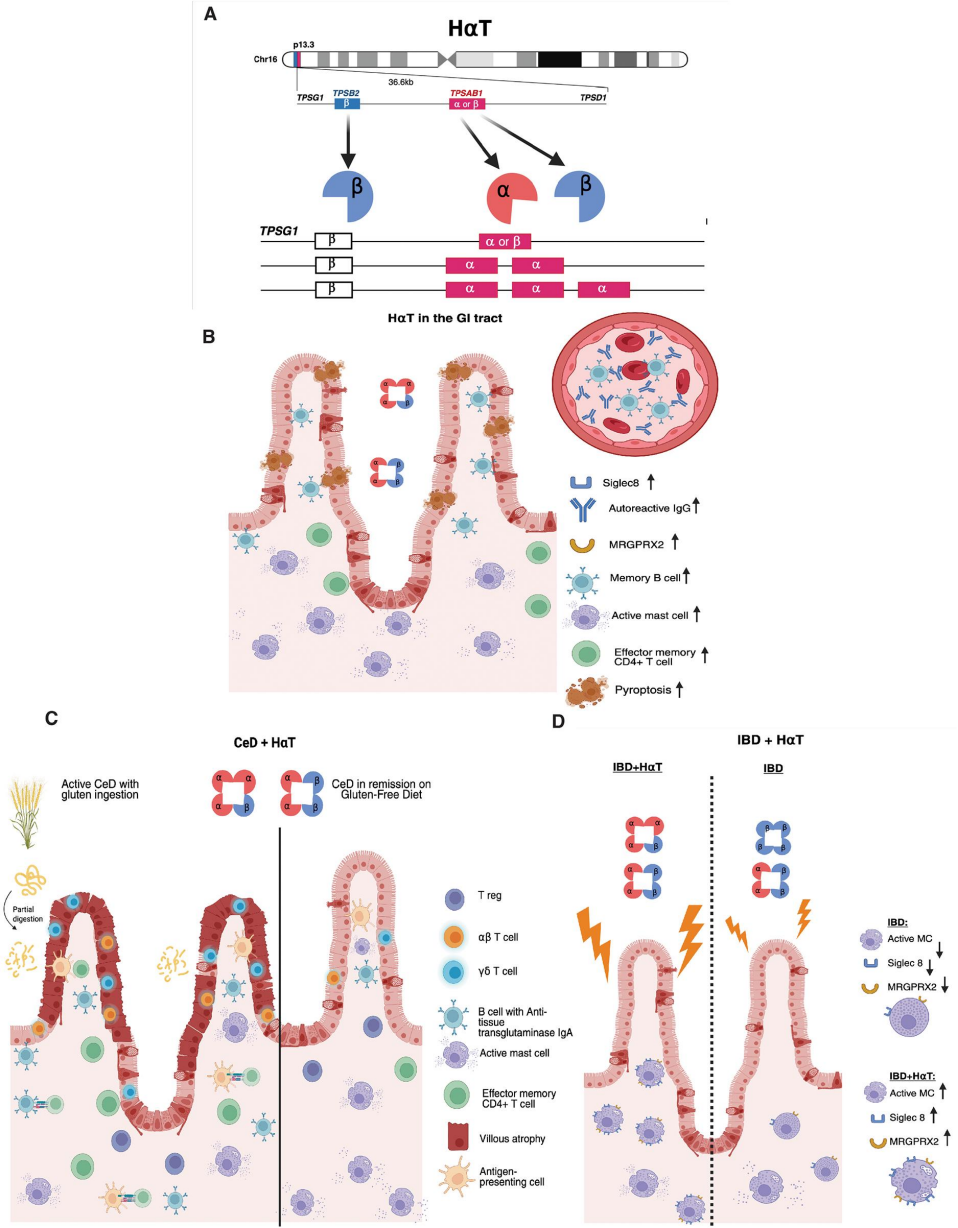
Hereditary alpha-tryptasemia genetics and impact on gastrointestinal health. **(A)** TPSAB1 is located in chromosome 16 and encodes for both, alpha and beta tryptase. Additional copies of this alelle lead to hereditary alpha-tryptasemia (HαT). **(B)** In HαT, the small intestine mucosa shows increased number of mast cells, higher autoreactive antibodies and higher memory B cells, leading to increased effector memory CD4+ T cells and pyroptosis. **(C)** In the presence of HαT in Celiac Disease, there are increased active mast cells and persistent symptoms despite a gluten-free diet. **(D)** The presence of HαT in Inflammatory bowel disease leads to more active mast cells and higher expression of Siglec 8 and MRGPRX2.

HαT is an autosomal dominant genetic trait caused by increased germline copy number of *TPSAB1*, which encodes both α- and β-tryptase (Figure 2A), and diagnosed via digital droplet Polymerase Chain Reaction (ddPCR) for tryptase genotyping (21, 22). *TPSAB1* contains either α- or β-tryptase; duplications (or, less commonly, triplications) of α-encoding alleles result in overproduction of α-protryptase and subsequent elevations in BST levels (14, 23–26). Each additional TPSAB1 copy increases BST by 9–10 ng/mL, reflecting a gene dosage effect (22, 23, 27, 28).

From a phenotypic standpoint, however, HαT is characterized by variable penetrance, symptoms may present at any age, and many carriers are asymptomatic (29). Among symptomatic individuals, commonly reported manifestations include cutaneous flushing or pruritus, gastrointestinal symptoms such as abdominal pain, bloating, or diarrhea, connective tissue and autonomic features, and an increased risk of anaphylaxis in some patients (22, 30, 31). To date, it is estimated that roughly one-third of individuals with this genetic trait are symptomatic. In this review, we define HαT by the presence of increased TPSAB1 copy number by ddPCR.

HαT affects approximately 4%–6% of individuals of European ancestry—equivalent to ∼20 million Americans (22, 23, 28)—and is reported much less frequently in other ancestral groups, though prevalence remains insufficiently defined. Human genomic datasets remain heavily skewed toward individuals of European ancestry, with non-European populations historically comprising a minority of participants in genome-wide association studies and related genomic analyses (32–34). As such, a major limitation of the current literature on hereditary α-tryptasemia (HαT) is the lack of ancestral diversity in studied cohorts. Most prevalence estimates derive from populations of European ancestry (22). Importantly, the presence of a genetic variant does not necessarily translate directly to phenotype, and genotype–phenotype relationships may differ across populations due to differences in genetic background, environmental exposures, and modifiers that influence MC biology and disease penetrance.

Current estimates may also be influenced by ascertainment bias, as many cohorts used to characterize HαT have been enriched for symptomatic individuals or patients referred for evaluation of MC–related disorders (35–38). Diagnostic access and referral patterns are not uniform across populations, and individuals from historically underrepresented or underserved groups may be less likely to undergo comprehensive genetic testing for MC disorders. As a result, both the prevalence of TPSAB1 copy number variation and the clinical spectrum associated with HαT may be incompletely captured in non-European populations. It is therefore possible that genetic variants associated with HαT may exist at comparable or even higher frequencies in other ancestral groups but remain underrecognized due to differences in clinical evaluation or reporting.

Expanding research to include ancestrally diverse populations will be critical for accurately defining the global distribution of TPSAB1 copy number variation and for refining genotype–phenotype relationships in HαT. Broader inclusion of diverse genomic datasets and population-based cohorts will help determine whether ancestry-specific modifiers influence symptom expression, MC–mediated disease risk, or the penetrance of HαT-associated traits. Such efforts will ultimately improve the generalizability of current findings and ensure that emerging diagnostic frameworks and clinical management strategies are applicable across diverse patient populations.

### 2.2 Mast cells, tryptase, and HαT

Several studies have demonstrated that HαT is associated with increased numbers of MCs in tissues. Initial studies focused on the bone marrow, where MCs were noted to be increased in number and appearance. Specifically, 5 patients (HαT+, c-kit D816V-) in the initial HαT cohort underwent a bone marrow biopsy which revealed a significant increase in MC numbers [9.6 cells/high-power field (hpf); range, 4–19.4 cells/hpf] vs. mean, 2.5 cells/hpf; range, 1–5 cells/hpf in healthy volunteers. MC aggregates and aberrant expression of CD2/CD25 were absent, helping to distinguish HαT-associated MC increases from clonal MC disorders such as systemic mastocytosis. Spindle-shaped MCs (>25%) were observed in a single patient (31). In a subsequent study, HαT bone marrow was associated with unique morphologic and histologic features when compared with controls. Specifically, MCs were larger, hypogranular, frequently detected in paratrabecular and perivascular locations, and associated with bone marrow eosinophilia (39).

In individuals with HαT, successive elevation of MCs in the human small intestine (duodenum and ileum) has been observed. MC numbers were found to correlate with elevated BST, and MCs expressed markers for activation and antigen presentation (40). An additional study, comparing MCs between individuals with and without HαT, confirmed that individuals with HαT had increased MCs in the duodenum (median = 30.0). These MCs were found in clusters and located throughout the mucosa and submucosa, including the superficial villi, compared to individuals without HαT. Spindle-shaped MCs were observed in all groups studied (38).

Taken together, these findings in the bone marrow and small intestine of individuals with HαT suggest that MCs play a key role in HαT symptomatology. This is most likely because MCs are key producers of tryptase (15). While several tryptases exist including *α*, *β*, *γ*, and *∂*, *α* and *β* are most significant in HαT because of their ability to form heterotetramers in MC granules and assemble into tetrameric protease complexes (7). In individuals with HαT, increased availability of α-tryptase promotes formation of *α*/*β* tryptase heterotetramers within MC granules. These heterotetramers exhibit greater enzymatic activity than β-tryptase homotetramers alone (14). Whereas α-tryptase alone lacks proteolytic activity, *α*/*β* heterotetramers can modulate MC function in an autocrine manner and activate tryptase-responsive pathways in neighboring cells, including neurons, epithelial cells, immune cells, and visceral smooth muscle (14, 41).

### 2.3 Heterotetramers and PAR2

*α*/*β* tryptase heterotetramers have unique functional properties and can trigger MC degranulation in response to vibration via cleavage of the EGF-like module-containing mucin-like hormone receptor-like 2 (EMR2) mechanosensory receptor. This mechanism underlies symptoms triggered by a mechanical stimulus (vibratory urticaria, pruritus, or pain) in patients with familial vibratory urticaria (42). *α*/*β* heterotetramers can also induce vascular leak via activation of Protease-activated receptor 2 (PAR2), a G protein coupled receptor (GPCR) present on neuron, smooth muscle, and endothelial cells among others (14, 17, 43). Unlike EMR2 activation, PAR2 activation occurs immediately upon its cleavage by *α*/*β* tryptase, potentially contributing to acute clinical phenotypes observed in MC activation such as hypotension, inflammation, pruritus, and hyperalgesia (14, 46, 47). It remains to be established if activation of PAR2 on neurons may play a mechanistic role in dysautonomia, another common feature of HαT. In the GI tract, PAR2 activation by *α*/*β* tryptase is associated with production of proinflammatory mediators (e.g., histamine, prostaglandins, leukotrienes, and cytokines) which collectively promote changes in mucosal permeability, visceral hypersensitivity, and motility (46, 47).

#### 2.3.1 Established data on role of PAR2 in barrier integrity, fibrosis, and nociceptive responses by neurons in the GI tract

The intestine is composed of distinct layers including the mucosa, submucosa, and muscularis propria. PAR2 has been shown to influence barrier integrity, tissue fibrosis and nociceptive responses by neurons in the GI tract and is widely distributed throughout the body (48, 49). PAR2 signaling plays an important role in the regulation of intestinal permeability, impacting gut barrier function under physiologic and pathologic conditions (50). Expressed in the apical and basolateral membrane of enterocytes, PAR2 regulates GI permeability via myosin light chain kinase (MLCK) and ß-arrestin (51). PAR2 is also expressed in endothelial cells, immune cells, and MCs. Under physiologic conditions, PAR2 activation enhances paracellular permeability (52). Increased expression of PAR2 is implicated in barrier dysfunction and may contribute to colonic inflammation (53).

Barrier dysfunction and fibrosis are key elements of intestinal disease. PAR2, in conjunction with COX2 and PPAR*γ*, is also implicated in tryptase-driven fibroblast proliferation and intestinal fibrosis (54). This pathway provides a mechanistic link between MC activity and fibrotic tissue remodeling, supporting the role of MCs in mediating both structural and inflammatory changes in intestinal pathology (55).

In the context of HαT, downstream effects of MC activation, tryptase release, and PAR2 activation include increased vascular permeability, smooth muscle hyperreactivity, neuroinflammation, and tissue remodeling (30). These consequences mediated by *α*/*β* heterotetramers provide a plausible molecular mechanism for HαT core clinical features including flushing/pruritus, systemic immediate hypersensitivity reactions, retained primary dentition, lower GI symptoms, and systemic venom reactions (30).

In addition to these tissue-level effects, PAR2 is also implicated in pain signaling. Activation of PAR2 on nociceptive sensory neurons sensitizes mechanosensitive ion channels such as transcript receptor potential vanilloid 4 (TRPV4). This results in enhanced release of neuropeptides, substance P (SP) and calcitonin gene-related peptide (CGRP), which mediate pain transmission (56). In the intestinal mucosa, tryptase from degranulated MCs can activate this pathway. Tryptase from degranulated MCs induces prolonged PAR2 activation which, if localized in proximity to colonic nerves, is implicated in pain and gut dysfunction observed in irritable bowel syndrome (IBS) (57, 58). Collectively, PAR2-mediated pathways driven by tryptase provide a mechanistic basis for the role of HαT as a disease modifier across various clinical contexts.

### 2.4 Hαt as a disease modifier: clinical associations and mechanistic considerations

HαT has been identified as a genetic modifier in several MC-associated diseases. HαT is associated with an increased risk for severe systemic anaphylaxis, most notably due to Hymenoptera venom (43, 59). Evidence from multiple studies suggests that higher BST levels are associated with more severe anaphylaxis (60–62). Among patients with clonal MC disorders, individuals with HαT more frequently report a history of anaphylaxis (35, 43, 63–65). Notably, HαT is enriched among patients with systemic mastocytosis (SM) and idiopathic anaphylaxis (43, 64). In these settings, increased α-tryptase gene dosage acts as a genetic modifier that amplifies MC activation severity. Furthermore, patients with MC activation symptoms and elevated BST have been reported to have unique bone marrow MC phenotypic and histologic changes (39). Even in the absence of clonal MC disease, HαT can account for elevated BST levels and may represent an independent risk factor for severe anaphylaxis (43). Selected studies investigating HαT in human cohorts and detailing clinical associations, genetic findings, and disease-modifying effects are summarized in Supplementary Table 1.

Clinical observations of HαT and MC-associated diseases raise an important mechanistic question regarding how increased TPSAB1 copy number influences MC biology and disease severity. Current evidence suggests that the primary consequence of additional TPSAB1 gene copies is increased production and secretion of α-tryptase, resulting in elevated BST concentrations rather than intrinsic expansion of MC populations (22, 23).

However, some human studies have reported tissue-level MC alterations in individuals with HαT, including increased MC density or distinct histologic features within gastrointestinal mucosa in patients with MC activation syndromes. These findings raise the possibility that sustained tryptase signaling may influence local MC recruitment, survival, or tissue remodeling in inflammatory microenvironments, although direct evidence that HαT drives MC proliferation remains limited (35, 36, 38).

From a mechanistic perspective, increased α-tryptase availability may primarily function as an amplifier of MC–dependent signaling pathways within tissues, rather than as a driver of MC expansion. In tissues such as the gastrointestinal mucosa, where MCs participate in neuroimmune communication and barrier regulation, sustained tryptase signaling may therefore contribute to altered inflammatory responses and local immune activation.

Because tryptase can also activate signaling pathways involved in neurovascular and inflammatory responses, increased BST associated with HαT has also been explored as a potential modifier of other disease processes. For example, HαT has been investigated in the context of postural orthostatic tachycardia syndrome (POTS); however, current evidence does not support a clear modifying role, although existing studies are limited by small cohort sizes (66, 67).

MCs have also been implicated in nociceptive pain pathways. Emerging data suggest that HαT is significantly enriched among fibromyalgia patients, compared to the general population. This association appears to involve co-inherited gain-of-function calcium channel variants of the gene *CACNA1H* (encoding CaV3.2). These variants have been implicated in individuals with HαT who exhibit chronic pain or fibromyalgia-like features, suggesting that neuronal signaling pathways may interact with MC biology to shape symptom profiles (68). Fibromyalgia exhibits clinical overlap with connective tissue disorders, which have also been studied in the context of HαT. Among Ehlers-Danlos Syndrome (EDS) patients, concurrent HαT diagnosis is associated with a unique endotype including dysphagia and retained primary dentition (36). However, larger studies will be necessary to determine HαT contribution to disease susceptibility or modification of clinical presentation in these disorders.

In addition, HαT has been shown to modify disease severity in patients with systemic mastocytosis (35, 64, 65, 69, 70), indicating that the presence of additional TPSAB1 copies can influence clinical outcomes (71). Collectively, these observations support a model in which TPSAB1 copy number variation interacts with additional genetic and molecular modifiers to generate the diverse clinical phenotypes observed in HαT.

#### 2.4.1 Immunopathology of the small intestine in symptomatic HαT in absence of other gastrointestinal conditions

We have shown that HαT is associated with increased intestinal epithelial cell pyroptosis, elevated MC density with markers of activation, and increased class-switched memory B cells in both the gut and peripheral blood even in patients without overt gastrointestinal diseases (38, 40, 72). These findings are consistent with a state of subclinical intestinal immune activation in some individuals with HαT (40). Individuals with HαT also have increased MC density across multiple tissue types, including the GI mucosa and bone marrow (38, 40).

These observations align with a broader body of literature demonstrating that MC–derived tryptases play important roles in regulating intestinal barrier function, epithelial stress responses, and neuroimmune signaling within the gastrointestinal mucosa (73). Tryptase signaling can influence epithelial integrity and inflammatory pathways within the gut, providing a potential mechanistic framework through which increased α-tryptase gene dosage may alter intestinal immune architecture (74, 75). In this context, increased MC density observed in gastrointestinal mucosa and other tissues in individuals with HαT may represent both a consequence and amplifier of tryptase-driven mucosal immune signaling. Collectively, these findings support a role for α-tryptase gene dosage in shaping mucosal immune architecture and inflammatory signaling pathways (Figure 2B**)**.

Furthermore, our group has shown that among individuals with HαT, GI tract auto-reactive IgGs are increased in the peripheral blood (40). This finding prompted investigation into the role of HαT as a potential disease modifier in celiac disease (CeD).

#### 2.4.2 Hαt is associated with persistent GI symptomatology in celiac disease

In addition to increased epithelial permeability by PAR2 and epithelial pyroptosis in the small intestine, MCs have been associated with celiac disease (CeD) pathogenesis (76). CeD is a common enteropathy associated with epithelial barrier dysfunction (80). In a well-defined cohort of 140 patients with CeD, 6.4% had coexisting HαT with elevated BST, mirroring its prevalence in the general population. In terms of intestinal pathology, duodenal MC counts were higher in individuals with CeD compared with controls (28). Among patients with both celiac disease and HαT, MC density was approximately 24% higher than in individuals with CeD alone (median 27.3 vs. 22.0 cells/high-power field; controls 18.4 cells/high-power field), although this difference did not reach statistical significance and should therefore be interpreted cautiously.

While HαT was not enriched in CeD, affected individuals were significantly more likely to experience persistent symptoms despite a gluten-free diet and normalized TTG IgA. Only one individual had persisting villous atrophy on their most recent duodenal biopsies, making superimposed ongoing gluten exposure an unlikely cause for these symptoms an increased number of duodenal MCs was observed in biopsies of patients with CeD and HαT with normal villous-crypt architecture (Figure 2C) (28). These findings suggest that HαT acts as a distinct disease process not associated with persisting villous atrophy, but contributing to symptoms which are a frequent reason for medical consultation in CeD (78).

There are many clinical and histopathologic similarities between CeD and HαT. Like CeD, HαT has variable manifestations, and many individuals with this trait may be asymptomatic (22, 29). Regarding intestinal pathology, HαT is associated with an increased frequency of MCs in the duodenum, especially in the epithelium, but also the lamina propria, muscularis, and submucosa (38). Interestingly, CeD and HαT also share features of epithelial disruption. Increased intestinal permeability may play a role in CeD onset, and early stage CeD is characterized by disruption of mucosal epithelial integrity (79, 80). We have also observed alterations in T cell subtype composition in CeD and HαT. Flow cytometric analyses of T cell subpopulations from duodenal biopsies of patients with CeD have demonstrated a 6–7-fold increase in the number of double-negative T cells (81). Examining small intestine of individuals with HαT alone, we have noted an increase in CD4 effector memory T cells (40). It is known that MCs contribute to recruitment of neutrophils, macrophages and T cells; they colocalize with T cells and play a role in their polarization, through secretion of either IFN-γ, TNF-α, IL-6 (82), IL-2 (83), leukotriene B4, or histamine (84). How these interactions affect CeD, with and without HαT, remains to be investigated. Moreover, some MC subtypes may be amplified depending on disease activity, as MC are also involved in mucosal healing, through release of FGF and FGF2, TGF-B1, VEGF and NGF (85). As such, there may be coexisting processes contributing to GI symptoms, and mucosal healing on a GFD in CeD with HαT.

#### 2.4.3 Hαt induces MC-driven inflammatory program in inflammatory bowel disease

After our prior observation of subclinical intestinal inflammation in individuals with HαT in the absence of GI comorbidities such as CeD and IBD (8, 40), we investigated MRGPRX2 in regards to emerging data on its role in IgE-independent MC activation and degranulation. Our published work compared HαT vs. non-HαT GI biopsies, where we observed that MRGPRX2 was upregulated in MCs from patients with HαT (in the absence of any coexisting GI disease) (Figure 2D) (86). CyTOF analysis of small intestinal biopsies from symptomatic (but histologically normal) individuals with HαT revealed an activated, MRGPRX2+ subset of MCs demonstrating increased expression of activation-associated markers including CD203c, LAMP-1, and Siglec8 (86); increased SIGLEC8 expression in HαT suggests a shift toward MC activation (87–89).

Examining MRGPRX2 in the context of IBD, MRGPRX2-mediated MC degranulation has been linked to active mucosal inflammation among patients with ulcerative colitis (UC) (90). Considering this finding and previous data linking MRGPRX2 with HαT-associated GI symptoms, our group examined MRGPRX2 in patients with HαT and IBD compared to patients with IBD alone. Performing spatial transcriptomics on descending colon tissue, we found that increased expression of MRGPRX2 mRNA in colon tissue from patients with HαT and IBD (UC and Crohn's) correlates with increased MC frequency in IBD patients with HαT (compared to samples from those with IBD alone) (86). While IBD inflammation in patients without HαT appears to result from a T-cell driven program, IBD with concomitant HαT is characterized by MC activation driven by MRGPRX2. Thus, MRGPRX2-induced MC activation may mediate GI symptoms in patients with HαT and IBD (Figure 2D) (86). The mechanisms underlying MRGPRX2-mediated effects may drive intestinal immune effects including barrier dysfunction, neuroimmune signaling, amplification of mucosal inflammation, and pseudoallergy (Supplementary Figure 1). HαT may therefore also be a disease modifier in intestinal inflammatory diseases.

## 3 Conclusion and future perspectives

Herein, we have reviewed data demonstrating that individuals with the HαT trait and core GI features of HαT, CeD, or IBD display altered immunopathology. As such, this data supports the hypothesis that HαT is a natural and distinctly informative *in vivo* overexpression model that is unique to humans and likely modifies disease behavior in the GI tract.

Several questions remain to be answered, including how α-tryptase gene dosage impacts MCs regionally along the GI tract. With the advent of modern cellular and molecular biology techniques such as single-cell sequencing, these questions may be further explored. It is also important to further determine how these tissue-level characteristics correspond to clinical manifestations. Patient-reported outcomes (PROs) provide valuable insights into symptom burden and quality of life but may not consistently reflect the underlying pathology, as symptom severity does not always correlate with objective measures of inflammation, tissue injury, or disease activity (91, 92). While it has previously been reported that HαT is not characterized by a certain clinical phenotype, we argue that it is important to examine core clinical features in large-scale real-world clinical datasets such as Epic Cosmos (93). Future studies integrating tissue-level molecular analyses with genotype and large-scale clinical phenotype data will be necessary to better understand how α-tryptase gene dosage affects intestinal biology. Epic Cosmos contains data from approximately 300 million patients across the United States, and the recent introduction of the ICD-10 code for HαT (D89.44) now allows identification of patients with this trait within the dataset. Approximately 4,322 individuals with HαT are currently represented; however, systematic analyses of these data are ongoing and results are not yet available. These approaches will also enable evaluation of HαT as a modifier of other conditions such as IBS and eosinophilic GI disorders. Given its high prevalence, HαT likely affects the presentation of other disorders in which MCs play a role. IBS is a prime candidate for future study given that MRGPRX2 signaling may be implicated in IBS, and the condition is common with high morbidity and few effective treatments (11).

Significant opportunities for drug repurposing and therapeutic development exist in the setting of MRGPRX2 and Siglec8 as therapeutic targets (94, 95). There is compelling clinical trial data on the use of a novel MRGPRX2 antagonist to treat MC-driven diseases (90). It is also important to understand how the effects of existing drugs, such as JAK-STAT inhibitors and anti-leukocyte trafficking drugs, may be mediated by the modulation of MC activity in the context of IBD. Understanding how variation in tryptase gene dosage impacts intestinal immunopathology will allow for continued innovation in the setting of MC-mediated diseases.

## Supplementary material

Supplementary Figure 1 **Proposed model linking HαT to MRGPRX2-mediated mast cellactivation in the intestinal mucosa.** Increased TPSAB1 copy number in HαT leads to elevated production of α-tryptase and increased BST levels. Enhanced tryptase availability contributes to heightened mast cell activation and may amplify local signaling pathways within tissue microenvironments. In the intestinal mucosa, this state is associated with increased expression and/or functional responsiveness of MRGPRX2 on mast cells. MRGPRX2 can be activated by a range of cationic ligands, including neuropeptides, antimicrobial peptides, and certain drugs, leading to non-IgE-mediated mast cell degranulation. This results in the release of mediators such as tryptase, histamine, cytokines, and proteases, which contribute to epithelial barrier dysfunction, neuroimmune signaling, and amplification of mucosal inflammation. Collectively, this model highlights a potential pathway by which HαT may enhance mast cell-driven intestinal immune responses through MRGPRX2-dependent mechanisms.
