# Supplementary figures and images for: Hereditary α-tryptasemia; a review of mechanisms linking α-tryptase gene dosage to intestinal homeostasis and immunopathology
Source: Front Allergy. 2026 Apr 14;7:1783914. doi: 10.3389/falgy.2026.1783914 (PMC13121348; doi:10.3389/falgy.2026.1783914)

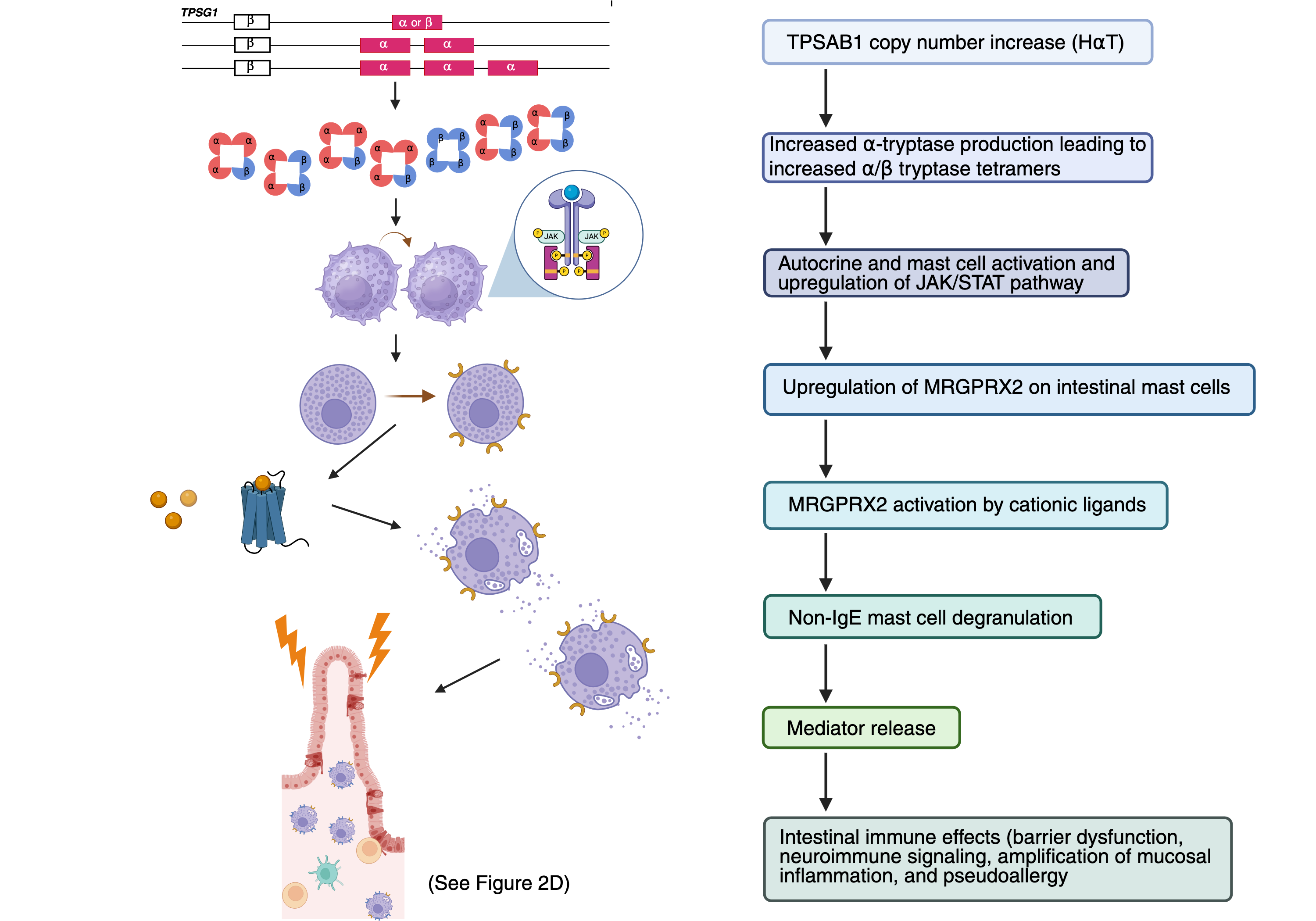

Supplement: Supplementary Figure 1 — Proposed model linking HαT to MRGPRX2-mediated mast cellactivation in the intestinal mucosa. Increased TPSAB1 copy number in HαT leads to elevated production of α-tryptase and increased BST levels. Enhanced tryptase availability contributes to heightened mast cell activation and may amplify local signaling pathways within tissue microenvironments. In the intestinal mucosa, this state is associated with increased expression and/or functional responsiveness of MRGPRX2 on mast cells. MRGPRX2 can be activated by a range of cationic ligands, including neuropeptides, antimicrobial peptides, and certain drugs, leading to non-IgE-mediated mast cell degranulation. This results in the release of mediators such as tryptase, histamine, cytokines, and proteases, which contribute to epithelial barrier dysfunction, neuroimmune signaling, and amplification of mucosal inflammation. Collectively, this model highlights a potential pathway by which HαT may enhance mast cell-driven intestinal immune responses through MRGPRX2-dependent mechanisms. [file Image1.png]
