# Supplementary material for: Hereditary α-tryptasemia; a review of mechanisms linking α-tryptase gene dosage to intestinal homeostasis and immunopathology
Source: Front Allergy. 2026 Apr 14;7:1783914. doi: 10.3389/falgy.2026.1783914 (PMC13121348; doi:10.3389/falgy.2026.1783914)
Supplement: Supplementary file 2 [file Table1.docx]

| **Citation** | **sample size** | **statistical analysis/power** | **Potential bias** | **study conclusions** |
| --- | --- | --- | --- | --- |
| 1.  González‐de‐Olano D, Navarro‐Navarro P, Muñoz‐González JI, et al. Clinical impact of the *TPSAB1* genotype in mast cell diseases: A REMA study in a cohort of 959 individuals. *Allergy*. 2023;79(3):711-723. doi:https://doi.org/10.1111/all.15911 | A total of 959 subjects were studied including 346 healthy donors (HD), 464 mastocytosis, and 149 non-clonal MCAS patients. | Large cohort: formal comparisons of HαT prevalence and clinical features; P-values reported (p=0.008, 0.018)Strong power for prevalence and subgroup analysis | Referral bias (mastocytosis/MCAS populations clinically referred); healthy donors may differ demographically; mixed disease subtypes | The frequency of HαT in MC disorders varies according to the diagnostic subtype of the disease. HαT does not imply a higher risk (and severity) of anaphylaxis in mastocytosis patients in whom anaphylaxis is not part of the presenting symptoms of the disease. |
| Greiner G, Sprinzl B, Górska A, et al. Hereditary α tryptasemia is a valid genetic biomarker for severe mediator-related symptoms in mastocytosis. *Blood*. 2021;137(2):238-247. doi:10.1182/blood.2020006157 | 180 mastocytosis + 180 controls + 720 other myeloid neoplasms + 61 validation cohort | ddPCR used for genotyping, group comparisons with P-values, independent validation cohort increases power for symptom association. Good power for prevalence and mediator outcomes; limited for some subgroups | Referral center, mastocytosis patients, controls sex-matched but not necessarily matched for other clinical confounder | HαT increases mediator symptoms and anaphylaxis risk in mastocytosis relative to controls |
| Polivka L, Madrange M, Bulai-Livideanu C, et al. Pathophysiologic implications of elevated prevalence of hereditary alpha-tryptasemia in all mastocytosis subtypes. *J Allergy Clin Immunol*. 2024;153(1):349-353.e4. doi:10.1016/j.jaci.2023.08.015 | 583 patients (556 mastocytosis + 27 Mastocycosis | Large cohort size (n=583) with 264 controls; group comparisons with P-values. Pooled  literature analysis to increase power for advSM comparison. Strong power for prevalence and symptom associations | Referral centered cohort (national mastocytosis center); retrospective design; mastocytosis subtypes unevenly sized | HαT is more prevalent in mastocytosis and linked with increased anaphylaxis risk; supports pathophysiologic involvement |
| Giannetti MP, Weller E, Bormans C, Novak P, Hamilton MJ, Castells M. Hereditary alpha-tryptasemia in 101 patients with mast cell activation-related symptomatology including anaphylaxis. *Ann Allergy Asthma Immunol*. 2021;126(6):655-660.doi:10.1016/j.anai.2021.01.016 | 101 HαT patients with mast-cell activation symptoms | Limited comparative analysis of allele copy number no reported power calculation, n=101 provides a moderate descriptive power but limited power for the genotype- phenotype or treatment analyses | Referral bias (mastocytosis centered population), Retrospective design, female skewed cohort (~80%), treatment response observational | HαT shows wide tryptase variability (>6.2 ng/mL) and multisystem mast cell activation symptoms; some severe manifestations (anaphylaxis) improve with omalizumab |
| Gülen T, Hägglund H, Dahlén B, Nilsson G. High prevalence of anaphylaxis in patients with systemic mastocytosis - a single-centre experience. *Clin Exp Allergy*. 2014;44(1):121-129. doi:10.1111/cea.12225 |  | 84 adult systemic mastocytosis patients |  |  |

**Supplemental Table 1.** **Selected studies examining hereditary α-tryptasemia (HαT) in human cohorts.** This table summarizes selected studies investigating HαT in human cohorts, including clinical associations, genetic findings, and disease-modifying effects. The studies included are intended to highlight key themes and representative findings in the field and do not constitute a comprehensive or systematic review of all published literature.
